# Chidamide triggers BTG1-mediated autophagy and reverses the chemotherapy resistance in the relapsed/refractory B-cell lymphoma

**DOI:** 10.1038/s41419-021-04187-5

**Published:** 2021-10-01

**Authors:** Kai Xue, Ji-Chuan Wu, Xi-Ya Li, Ran Li, Qun-ling Zhang, Jin-Jia Chang, Yi-Zhen Liu, Chun-Hui Xu, Jia-Ying Zhang, Xiao-Jian Sun, Juan J. Gu, Wei-Jian Guo, Lan Wang

**Affiliations:** 1 Shanghai Institute of Hematology, State Key Laboratory of Medical Genomics, National Research Center for Translational Medicine at Shanghai, Ruijin Hospital Affiliated to Shanghai Jiao Tong University School of Medicine, Shanghai, China; 2 Department of Medical Oncology, Fudan University Shanghai Cancer Center, Shanghai, 200032 China; 3 CAS Key Laboratory of Tissue Microenvironment and Tumor, Shanghai Institute of Nutrition and Health, Shanghai Institutes for Biological Sciences, University of Chinese Academy of Sciences, Chinese Academy of Sciences, Shanghai, 200031 China; 4 Department of Medicine & Immunology, Roswell Park Comprehensive Cancer Center, Buffalo, NY USA

**Keywords:** Targeted therapies, B-cell lymphoma

## Abstract

Rituximab/chemotherapy relapsed and refractory B cell lymphoma patients have a poor overall prognosis, and it is urgent to develop novel drugs for improving the therapy outcomes. Here, we examined the therapeutic effects of chidamide, a new histone deacetylase (HDAC) inhibitor, on the cell and mouse models of rituximab/chemotherapy resistant B-cell lymphoma. In Raji-4RH/RL-4RH cells, the rituximab/chemotherapy resistant B-cell lymphoma cell lines (RRCL), chidamide treatment induced growth inhibition and G0/G1 cell cycle arrest. The primary B-cell lymphoma cells from Rituximab/chemotherapy relapsed patients were sensitive to chidamide. Interestingly, chidamide triggered the cell death with the activation of autophagy in RRCLs, likely due to the lack of the pro-apoptotic proteins. Based on the RNA-seq and chromatin immunoprecipitation (ChIP) analysis, we identified *BTG1* and *FOXO1* as chidamide target genes, which control the autophagy and the cell cycle, respectively. Moreover, the combination of chidamide with the chemotherapy drug cisplatin increased growth inhibition on the RRCL in a synergistic manner, and significantly reduced the tumor burden of a mouse lymphoma model established with engraftment of RRCL. Taken together, these results provide a theoretic and mechanistic basis for further evaluation of the chidamide-based treatment in rituximab/chemotherapy relapsed and refractory B-cell lymphoma patients.

## Introduction

Currently, the standard first-line treatment for patients with diffuse large B-cell lymphoma (DLBCL) is rituximab combined with chemotherapy, which leads to an around 60% complete remission rate. Despite overall improvements in the clinical outcomes of DLBCL, approximately one-third of patients still develop relapsed/refractory disease. The clinical approach to relapsed/refractory DLBCL include high-dose chemotherapy and autologous stem cell transplantation (HD-ASCT). However, the patients refractory to rituximab have poor outcomes with HD-ASCT [1]. Thus, there is an urgent need for new drugs to improve the outcomes of the salvage therapies [2, 3].

The rituximab resistance cell lines (Raji-4RH and RL-4RH) were developed by repeated exposure to rituximab, and these rituximab/chemotherapy resistant B-cell lymphoma cell lines (RRCLs) also displayed significant resistance to a variety of chemotherapy drugs. RRCL has been used as an excellent pre-clinical model to evaluate the biological activity of newly developed drug. The RRCLs show decreased CD20 cell surface marker, which is the target of rituximab. This decrease of CD20 is because of a reduction of positive regulatory proteins binding to CD20 promoter and a defect in CD20 transport to the cell surface [4]. Furthermore, RRCLs carry many defects in the BCL-2 family members, such as silence of the pro-apoptotic proteins Bak and Bax [5, 6], which makes RRCLs not undergo rituximab/chemotherapy-induced apoptosis. Given the deficiencies of the pro-apoptotic proteins, the therapeutic strategy targeting the autophagy pathway could be effective in the rituximab/chemotherapy relapsed and refractory B-cell lymphoma. Thus, it is worth testing the therapeutic efficacy of a novel histone deacetylase (HDAC) inhibitor, chidamide, in the RRCL-derived cell/mouse model, which will be helpful for the future clinical application of HDAC inhibitor in the treatment of relapsed/refractory B-cell lymphoma.

Chidamide is a selective HDAC inhibitor of benzamide class developed in China, and it is able to promote histone H3 acetylation [7, 8]. Recently, it has been reported that chidamide could block the differentiation and resorption of osteoclast [9]. Clinical studies showed that low-dose chidamide administration restores immune tolerance in immune thrombocytopenia, which suggests that chidamide is low-toxic and safe in clinic [10]. Chidamide inhibits cell proliferation and induces cell apoptosis in different types of hematological malignancies [11], [12], [13–15]. The combination of chidamide and decitabine, a hypomethylating agent inhibited the growth of p300- or KMT2D-mutated T-lymphoma cells [16]. Moreover, clinical studies showed that chidamide had a favorable efficacy for angioimmunoblastic T-cell lymphoma [17] and relapsed/refractory peripheral T-cell lymphoma patients [18, 19]. However, it has not been determined whether chidamide, either as a single agent or as a component of combined therapy, could induce autophagy in the rituximab/chemotherapy relapsed and refractory B-cell lymphoma cells.

## Materials and methods

### Cell lines

A panel of RSCL (the rituximab sensitive cell lines) and RRCL were used in this experiment. The RSCL Raji [Burkitt’s lymphoma (BL)], RL [germinal center B cell (GCB) DLBCL], and the RRCL (Raji-4RH and RL-4RH) were kind gifts given from Czuczman. RRCL were created and characterized from RSCL as previously described [20]. All cell lines were maintained in RPMI 1640 with Glutamax-1 (Gibco, C11875500CP) supplemented with 10% heat-inactivated fetal bovine serum (FBS), HEPES (5 mmol/L), penicillin and streptomycin (100 IU/mL), and sodium pyruvate (1 mmol/L).

### Primary patient samples

Primary relapsed patient cells were obtained from puncture samples from consenting patients with rituximab or chemotherapy treatment failure. Patient #1 was a 57-year-old man who had become resistant from 6 times rituximab plus CHOP treatment; patient #2 was a 63-year-old man who was diagnosed as non-hodgkin’s lymphoma; patient #3 was a DLBCL patient who had relapsed from 3 times rituximab plus CHOP treatment. All primary patient cells were cultured at 37 °C in RPMI 1640 supplemented with 20% FBS and penicillin/streptomycin (100 IU/mL). Informed consent was obtained from all subjects for the collection and use of samples.

### Mouse model and drugs administration

2.5 × 10^6^ Raji-4RH cells were suspended in RPMI medium and mixed with 35 µL matrigel to 100 µL. The cells were injected subcutaneously into both flanks of nude mice (5 or 6-week-old). Treatment started when tumor became about 5 × 5 mm in surface (day 0). Animals were randomly divided into 4 groups (6 mice/group) to receive control, chidamide (25 mg/kg), cisplatin (1 mg/kg), and chidmaide + cisplatin treatment, respectively, for 16 days. Chidamide was given intragastric administration in the morning and cisplatin was given by intraperitoneal injection in the afternoon. Tumor volumes were calculated as 0.5 × *a* × *b*^2^, where ‘*a*’ is the length and ‘*b*’ is the width. Approval number of committees for ethical review is SINH-2021-WL-3.

### Methods used

Measurement of cell viability assay, isobolographic analysis, lentivirus production and infection, shRNA-mediated gene knockdown, RT-PCR and RT-qPCR, ChIP-qPCR, RNA-seq, flow cytometry, and statistical analyses are provided in the supplementary method section.

## Results

### Chidamide induced growth inhibition and cell cycle arrest in RRCL

**Fig. 1:**
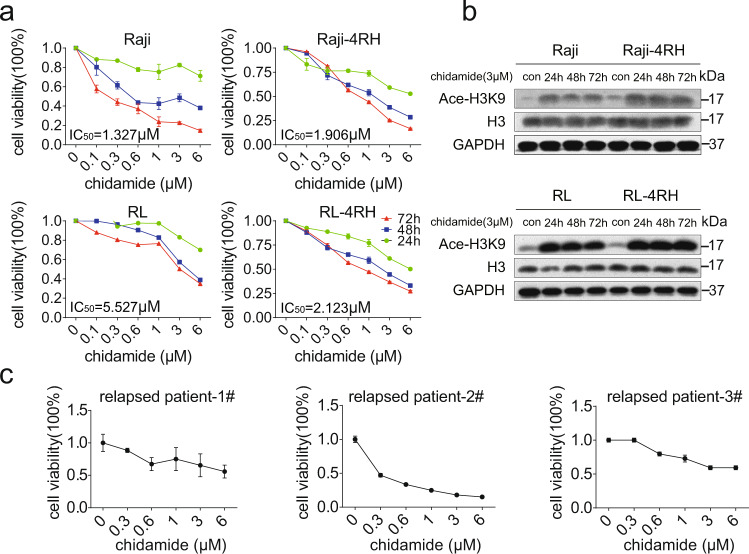
Chidamide treatment reduced cell viability of RSCL, RRCL, and primary relapsed B-cell lymphoma cells. **a** Raji, Raji-4RH, RL, and RL-4RH were treated with chidamide at different time points and concentrations. Cell viability were measured by using MTT assay, and IC50 of chidamide were calculated (*N* = 3). **b** Acetylation of histone H3 lysine 9 (H3K9) was examined by using Western blot assay 24, 48 or 72 h after chidamide (3 μM) treatment (*N* = 3). **c** The primary relapsed B-cell lymphoma cells were treated with chidamide at different concentrations for 24 h (#1) or 72 h (#2 and #3). Cell viability was measured by using MTT assay (*N* = 3). For all graphs, data are presented as mean ± SD.

**Fig. 2:**
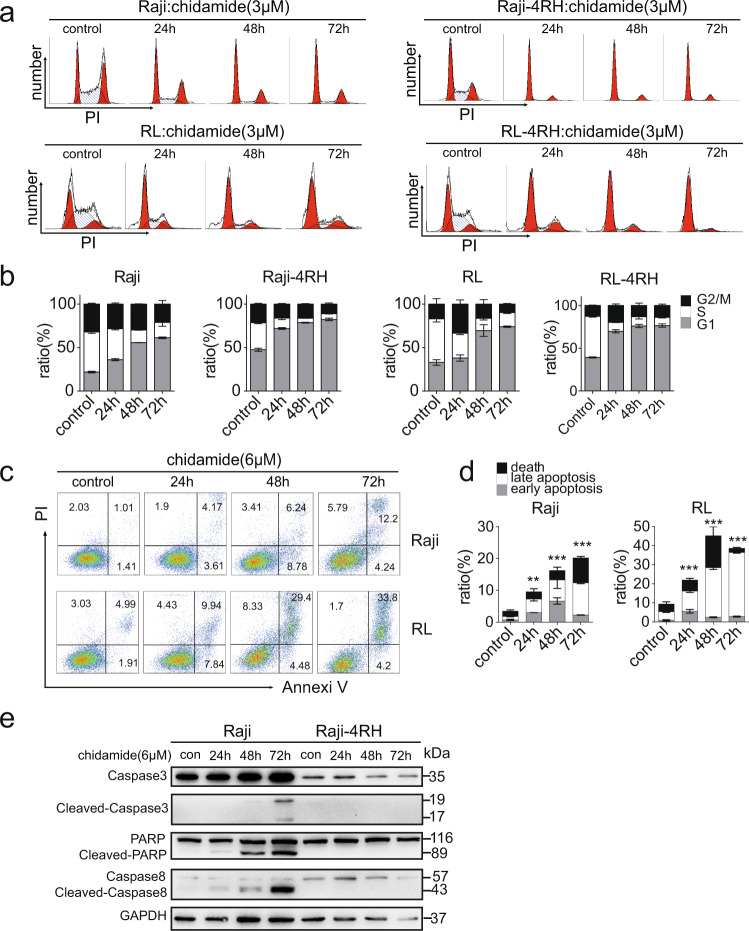
Chidamide treatment induced cell cycle arrest in both RSCL and RRCL, but triggered cell apoptosis only in RSCL. **a**, **b** The cell cycle of RSCL and RRCL treated with chidamide (3 μM) for 24, 48 or 72 h were examined by flow cytometry analyses (**a**). The ratio of G1, S, and G2/M phase were shown in histograph (**b**). **c**, **d** Apoptosis of RSCL induced by chidamide (6 μM) treatment for 24, 48, or 72 h were examined by using flow cytometry analysis (**c**). The statistics of early apoptosis, late apoptosis, and cell death were shown in corresponding histograph (**d**). **e** Changes of apoptosis related proteins were examined by using Western blot analysis in Raji and Raji-4RH treated with chidamide (6 μM) or DMSO for 24, 48, and 72 h (*N* = 3). For all graphs, data are presented as mean ± SD.

Raji, Raji-4RH, RL, and RL-4RH cells were treated with chidamide at various dosages for 72 h, and the cell viability were examined at different time points. Chidamide inhibited the growth of both Rituximab sensitive cells (Raji and RL) and Rituximab resistant cells (Raji-4RH and RL-4RH) in a dose- and time-dependent manner. The IC50 of chidamide in Raji, Raji-4RH, RL, and RL-4RH cells are 1.327, 1.906, 5.527, and 2.123 μM, respectively (Fig. 1a). As an HDAC inhibitor, chidamide significantly increased the acetylation level of Histone H3 lysine (H3K9) in these rituximab-resistant and -sensitive cells (Fig. 1b and Fig. S1). Moreover, the primary B-cell lymphoma cells from relapsed patients were also sensitive to chidamide treatment (Fig. 1c). Next, we performed flow cytometric analysis of cell cycle and cell apoptosis in these cells treated with chidamide or the vehicle control. We found that 1, 3, or 6 μM chidamide treatment resulted in the significant G0/G1 phase cell cycle arrest in both RRCL and RSCL (Fig. 2a, b and Fig. S2a, b). The Annexin V/PI staining analysis showed that chidamide induced apoptosis in Raji and RL cells (Fig. 2c, d). However, chidamide could not induce apoptosis in Raji-4RH and RL-4RH cells (Fig. S2c, S2d). Next, we evaluated the level of apoptosis-related proteins and found that chidamide could not trigger the cleavage of Caspase-3, PARP, and Caspase-8 (Fig. 2e), in Raji-4RH and RL-4RH cells, which was possibly due to the lack of pro-apoptotic proteins in the RRCL [5, 6]. Therefore, these results suggested that the cell cycle arrest but not apoptosis was involved in chidamide-induced cell growth inhibition in RRCL.

### The integrated gene expression profiling of RRCL following chidamide treatment

**Fig. 3:**
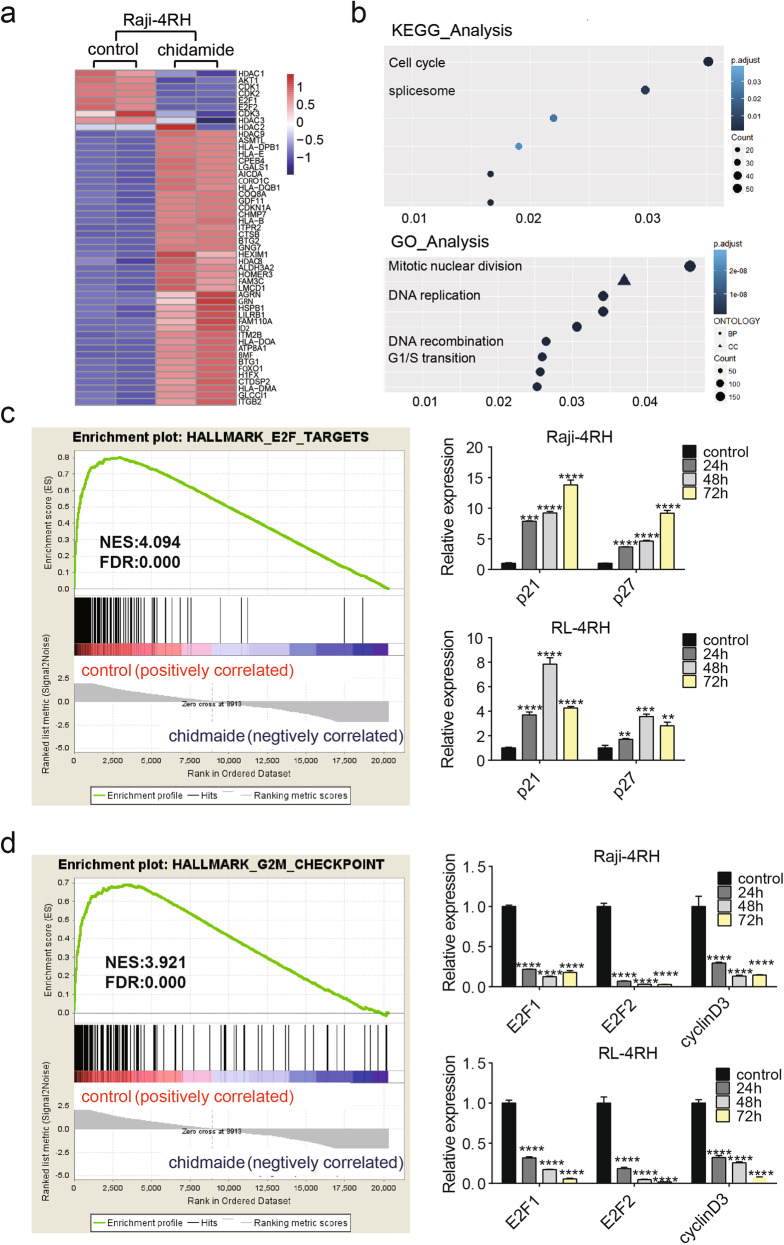
Cell cycle arrest molecular signature induced by chidamide treatment. **a** Heatmap of differentially expressed gene in the RNA-seq analysis of Raji-4RH treated with chidamide (3 μM) for 24 h (*N* = 3). **b** KEGG pathway and GO analysis revealed top molecular functions affected by chidamide treatment are cell cycle related (*N* = 3). **c**, **d** GSEA analysis of all genes shows a downregulation in HALLMARK E2F TARGETS genset after chidamide treatment compared with the control, and the expression of p21/p27 was detected by performing Q-PCR analysis 24, 48, or 72 h after chidamide treatment (**c**); downregulation in HALLMARK G2/M CHECKPOINT after chidamide treatment compared with the control, and the expression of E2F1, E2F2, and cyclinD3 was examined by using Q-PCR analysis 24, 48, or 72 h after chidamide treatment (**d**). For all graphs, data are presented as mean ± SD, *p < 0.05, **p < 0.01, *p* < 0.005***, *p* < 0.001****. Statistical analysis was performed with a paired *t* test.

**Fig. 4:**
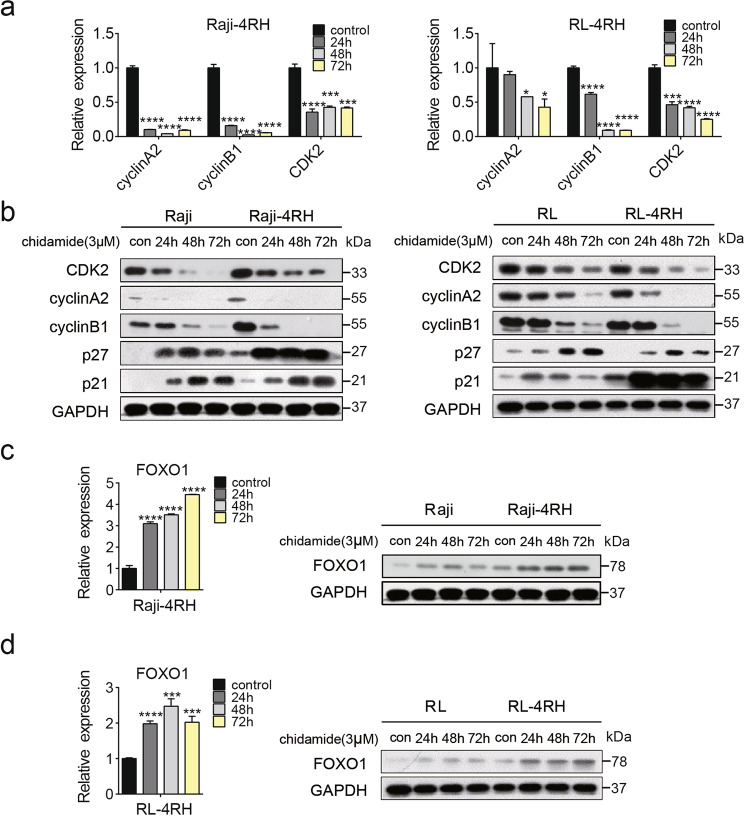
Upregulation of FOXO1 and changes of cell cycle-related proteins induced by chidamide treatment. **a**, **b** Changes of p21, p27, CyclinA2, CDK2, and CyclinB1 were examined by Q-PCR and Western blot analysis in RRCL treated with chidamide (3 μM) for 24, 48 or 72 h (*N* = 3). **c**, **d** Changes of FOXO1 were examined by Q-PCR and Western blot in RRCL treated with chidamide (3 μM) for 24, 48, or 72 h (*N* = 3). For all graphs, data are presented as mean ± SD, *p < 0.05, **p < 0.01, *p* < 0.005***, *p* < 0.001****. Statistical analysis was performed with a paired *t* test.

To identify the novel target genes and pathways regulated by chidamide to promote the death of RRCL, we treated Raji-4RH cells with 3 μM chidamide or the control vehicle for 24 h, and the RNA of these cells were isolated for RNA-seq analysis. The heat map analysis showed some differentially expressed genes influenced by chidamide in Raji-4RH cells, including those that regulate the cell cycle pathway (Fig. 3a). We also performed KEGG pathway and GO analysis for the RNA-Seq data, and the top molecular functions influenced by chidamide treatment were related to cell cycle (Fig. 3b). We also analyzed the entire unfiltered expression dataset with the Gene Set Enrichment Analysis (GSEA) tool, using the Molecular Signatures Database. GSEA identified significant sets of genes that were overrepresented at the top or bottom of the ranked set of differentially expressed genes comparing the control and the chidamide treated Raji-4RH cells. GSEA curves for enriched pathways involving E2F targets (Fig. 3c left panel) and G2/M checkpoint pathways (Fig. 3d left panel). To define the expression of cell cycle regulators in the E2F and G2/M checkpoint pathways that may be modulated by chidamide, we examined Raji, Raji-4RH, RL, and RL-4RH cells treated with 3 μM chidamide for 24, 48, or 72 h. Chidamide treatment upregulated the amount of p21 and p27 mRNA (Fig. 3c right panel and Fig. S3a), and downregulated the mRNA level of E2F1, E2F2, and cyclin D3 in Raji-4RH and RL-4RH cells (Fig. 4d right panel and Fig. S3b). Thus, chidamide appeared to induce the cell cycle arrest of RRCL through these targets within the E2F and G2/M checkpoint pathways.

### Chidamide-induced cell growth inhibition and cell cycle arrest can be rescued by inhibition of FOXO1 in RRCL

**Fig. 5:**
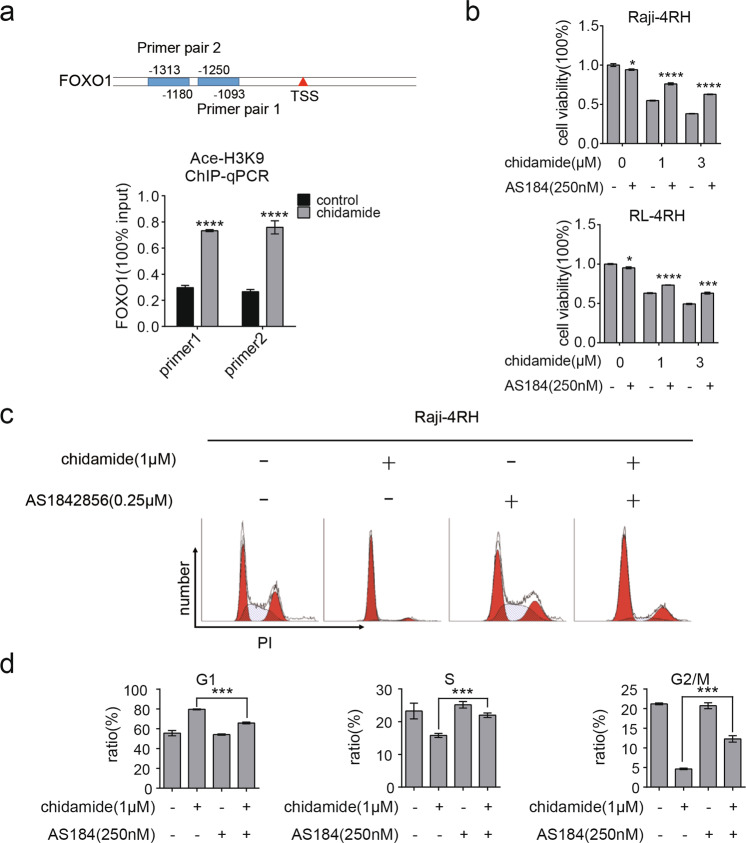
FOXO1 was a target gene of chidamide to mediate cell cycle arrest. **a** Raji-4RH was treated with chidamide (3 μM) or DMSO for 24 h and then cells were collected for ChIP assay. DNA fragments were pulled down with Ace-H3K9 antibody or negative IgG antibody. Enrichment of *FOXO1* promoter sequences were examined by Q-PCR analysis and normalized to input chromatin DNA, and primers were designed as the sketch indicated (*N* = 3). **b** Raji-4RH and RL-4RH cells were treated with AS184 (250 nM) and chidamide (1 or 3 μM) alone or in combinations for 48 h, and cell viability were examined by MTT assay (*N* = 3). **c**, **d** Raji-4RH and RL-4RH cells were treated with AS184 (250 nM) and chidamide (1 μM) alone or in combinations for 48 h, and cell cycle were examined by flow cytometry (**c**) and shown in histograph (*N* = 3) (**d**). For all graphs, data are presented as mean ± SD, *p < 0.05, **p < 0.01, *p* < 0.005***, *p* < 0.001****. Statistical analysis was performed with a paired *t* test.

We have identified some cell cycle-related genes influenced by chidamide in the above RNA-Seq analysis. To further confirm the RNA-seq data, we performed Q-PCR and Western blot analyses in Raji, Raji-4RH, RL, and RL-4RH cells treated with 3 μM chidamide for 24, 48, or 72 h. Cyclin A2, CDK2, and Cyclin B1 were decreased and p21/p27 were increased at both mRNA and protein levels upon the chidamide treatment (Fig. 4a, b and Fig. S4a, b). We also found that the chidamide treatment upregulated the expression of FOXO1, the key upstream regulator of cell cycle, in Raji, Raji-4RH, RL, and RL-4RH cells (Fig. 4c, d and Fig. S4c, d). To understand the regulatory mechanism, we performed the ChIP assay using the antibody to the acetylated histone H3K9 in Raji-4RH cells treated with 3 μM chidamide or the control vehicle for 24 h. A significant increase in acetylated H3K9 on the promoter region of *FOXO1* was detected in the Raji-4RH cells treated with chidamide compared with levels in the controls (Fig. 5a), which could explain the chidamide-induced upregulation of *FOXO1* gene expression.

To determine whether FOXO1 mediated the effects of chidamide on the growth inhibition and cell cycle arrest, we performed the rescue assay with FOXO1 inhibition. As shown in Fig. 5b, the decreased cell viability in the chidamide-treated Raji-4RH and RL-4RH cells were attenuated by using a small-molecule inhibitor of FOXO1 (AS184). FOXO1 inhibitor also rescued the G0/G1 phase cell cycle arrest in the Raji-4RH and RL-4RH cells treated with chidamide (Fig. 5c, d). These results suggested that chidamide might impair the growth and cell cycle of RRCL by upregulating FOXO1.

### Chidamide induced the death of RRCL and BTG1-mediated autophagy

**Fig. 6:**
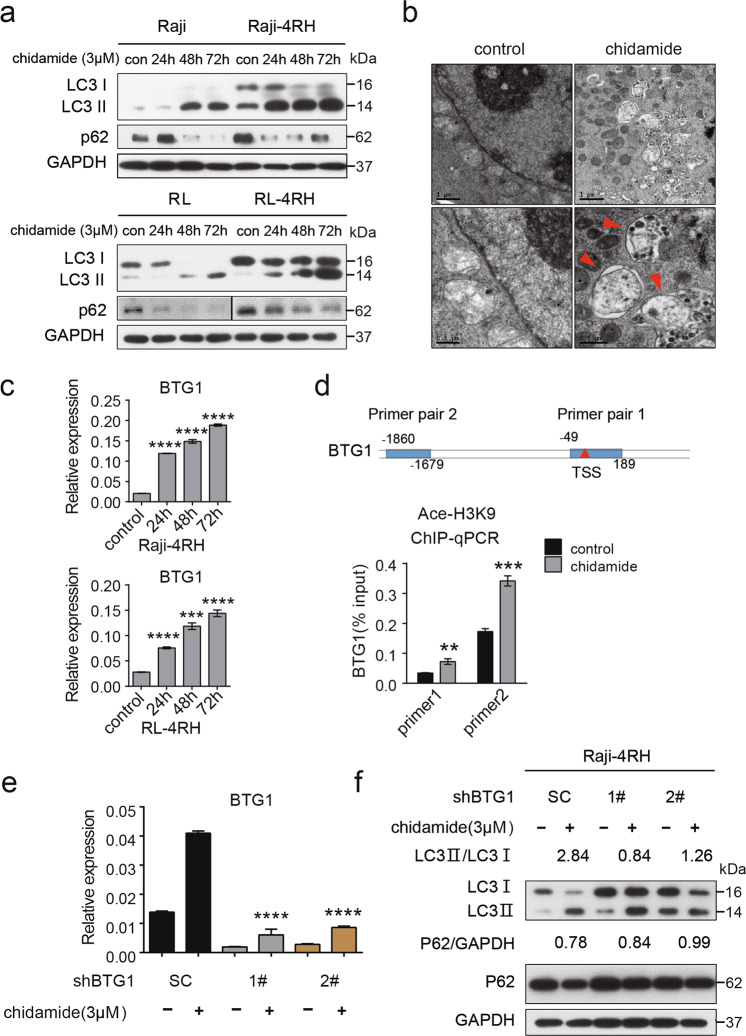
Chidamide treatment induced robust autophagy through upregulation of BTG1. **a** RSCL and RRCL were treated with DMSO or chidamide (3 μM) for 24, 48, or 72 h and autophagy-related proteins were detected by using Western blot analysis (*N* = 3). **b** More autophagosomes of Raji-4RH were detected in Raji-4RH treated with chidamide (3 μM) compared with the DMSO control, red arrows indicates autophagosomes observed in electron microscope (*N* = 1). **c** Expression of BTG1 in Raji-4RH and RL-4RH treated with chidamide (3 μM) for 24, 48, or 72 h were detected by Q-PCR (*N* = 3). **d** Raji-4RH was treated with chidamide (3 μM) or DMSO for 24 h and then cells were collected for ChIP assay. DNA fragments were pulled down with Ace-H3K9 antibody or negative IgG antibody. Enrichment of BTG1 promoter sequences were examined by Q-PCR and normalized to input chromatin DNA, and primers were designed as the sketch indicated (*N* = 3). **e** Efficiency of BTG1 knock down with shRNA before and after chidamide treatment were examined by using Q-PCR analysis (*N* = 3). **f** ShRNA SC, shRNA#1 and #2 Raji-4RH were treated with chidamide (3 μM) for 48 h, and then autophagy-related proteins LC3-II, LC3-I, and p62 were examined by using Western blot analysis (*N* = 3). SC Scramble. For all graphs, data are presented as mean ± SD, *p < 0.05, **p < 0.01, *p* < 0.005***, *p* < 0.001****. Statistical analysis was performed with a paired *t* test.

To explore the mechanism of chidamide-induced RRCL death, we evaluated the protein level of p62, an autophagy biomarker, and found increased autophagic activity manifested by decreased p62 protein levels in Raji, Raji-4RH, RL, and RL-4RH cells treated with chidamide for 24, 48, or 72 h (Fig. 6a and Fig. S5a). LC3-postitive double-membrane degradation cargo is also considered to be the hallmark of autophagy activity [21]. Therefore, we measured the conversion of LC3-I to LC3-II and LC3 autophagic vesicles. Increased LC3-II was observed in Raji, Raji-4RH, RL, and RL-4RH cells upon chidamide treatment, suggesting more conversion of LC3-I to LC3-II (Fig. 6a and Fig. S5a). We found that chidamide treatment in Raji-4RH cells increased the number of autophagic vesicles through electron microscope (Fig. 6b).

We treated Raji, Raji- 4RH, RL, and RL- 4RH cells with the autophagy inhibitor, Bafilomycin A1, and performed the Western blotting analysis, which showed that the combination of chidamide and Bafilomycin A1 increased levels of LC3 II and p62 compared with the chidamide single treatment controls (Fig. S5b). After transfected with EGFP-LC3B, the Raji- 4RH and RL- 4RH cells treated with the combination of chidamide and Bafilomycin A1 showed stronger signals than chidamide single treatment controls (Fig. S5c), and the Raji- 4RH and RL- 4RH cells treated with chidamide showed dramatically increased free GFP fragments (Fig. S5d). These data suggested that chidamide increased autophagy induction in RRCL.

To understand the underlying mechanism, we analyzed the RNA-Seq data, and found that chidamide treatment upregulated the expression of BTG1 (B-cell translocation gene 1), a key regulator of autophagy [22, 23], in Raji-4RH cells. The Q-PCR analysis confirmed that chidamide induced the upregulation of BTG1 in Raji-4RH and RL-4RH cells (Fig. 6c). Then, we performed the ChIP assay, and the results showed that the binding of acetyl histone H3K9 to *BTG1* promoter to acetyl histone H3K9 was significantly increased by chidamide treatment (Fig. 6d), suggesting that *BTG1* could be a target gene of chidamide. To examine the role of BTG1 in chidamide-induced autophagy, we performed the rescue assay with BTG1 inhibition. The increased cell autophagy in the chidamide-treated Raji-4RH cells were attenuated by using the shRNA against BTG1. Knockdown of BTG1 rescued the conversion of LC3-I to LC3-II in the Raji-4RH cells treated with chidamide (Fig. 6e, f). The flow analysis showed that knockdown of BTG1 could not induce apoptosis in Raji-4RH and RL-4RH cells (Fig. S5e). These results indicated that chidamide promoted the autophagy of RRCL by upregulating BTG1.

### Chidamide in combination with chemotherapy drugs showed synergistic effect in RRCL and significantly inhibited the growth of rituximab-resistant lymphoma in vivo

**Fig. 7:**
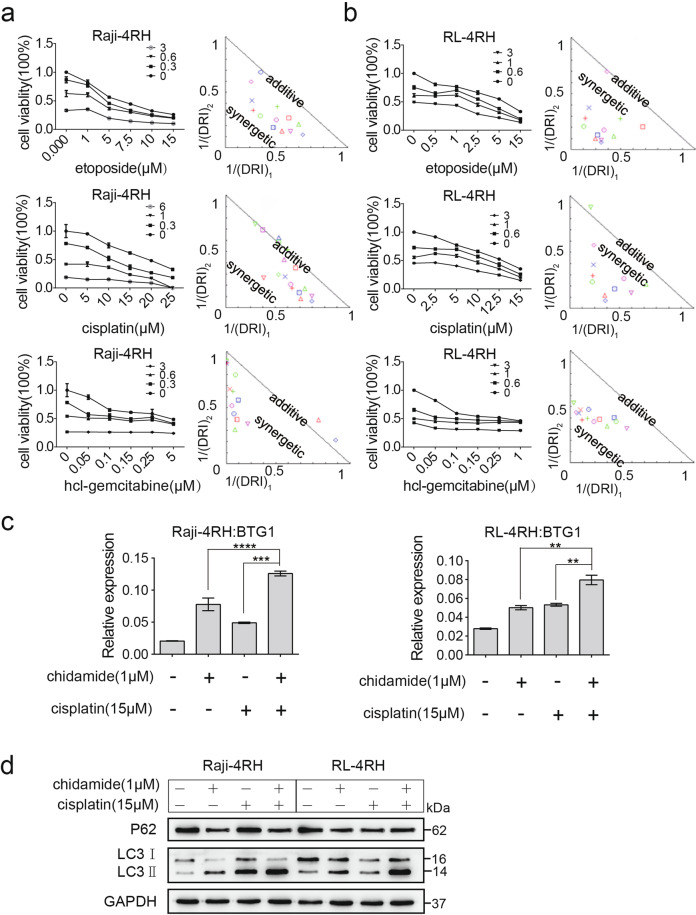
Chidamide and chemotherapeutics were synergetic in vitro through congenerous upregulation of BTG1. **a**, **b** Raji-4RH (**a**) and RL-4RH (**b**) were treated with the different combinations of chidamide and etoposide, cisplatin, hcl-gemcitabine for 72 h and cell viability were examined by using MTT assay. Cooperativity of chidamide and chemotherapeutics were calculated by Compusyn software. The normalized isobologram were shown. DRI: Dose-Reduction Index (*N* = 3). **c** The expression of BTG1 in Raji-4RH and RL-4RH treated with DMSO, chidamide (1 μM), cisplatin (15 μM) or their combination for 48 h were examined by Q-PCR (*N* = 3). **d** Changes of autophagy-related proteins in Raji-4RH and RL-4RH treated with DMSO, chidamide (1 μM), cisplatin (15 μM) or their combination for 48 h were examined by using Western blot analysis (*N* = 3). For all graphs, data are presented as mean ± SD, *p < 0.05, **p < 0.01, *p* < 0.005***, *p* < 0.001****. Statistical analysis was performed with a paired *t* test.

Raji, Raji-4RH, RL, and RL-4RH cells were treated with the combination of chidamide and the chemotherapy drugs, including cisplatin, etoposide, hcl-gemcitabine, and doxorubicin at different dosages for 72 h. The combinative effects of these drugs were examined by using MTT assay, which provided the basis for making the synergistic curves. Chidamide at 0.3 and 0.6 μM in combination with cisplatin, etoposide or hcl-germcitabine showed synergistic effect in the Rituximab resistant Raji-4RH and RL-4RH cells (Fig. 7a, b and Fig. S6a, b). However, the combination of chidamide and Dox showed certain antagonistic effects at some combinative dosages (Fig. S6c). Next, we focused on the combination of chidamide and cisplatin, which was found to induce more upregulation of BTG-1 compared with chidamide/cisplation single treatment control in Raji-4RH and RL-4RH cells (Fig.7c). In addition, the combination of chidamide and cisplatin treatment also resulted in the increased LC3-II and pH2A.X levels and the decreased Rad51 level compared with chidamide or cisplation single treatment control in Raji-4RH and RL-4RH cells (Fig. 7d and Fig. S6d). These results suggested that cisplatin could be the suitable candidate drug for the combination treatment of chidamide in rituximab-resistant B-cell lymphoma.

**Fig. 8:**
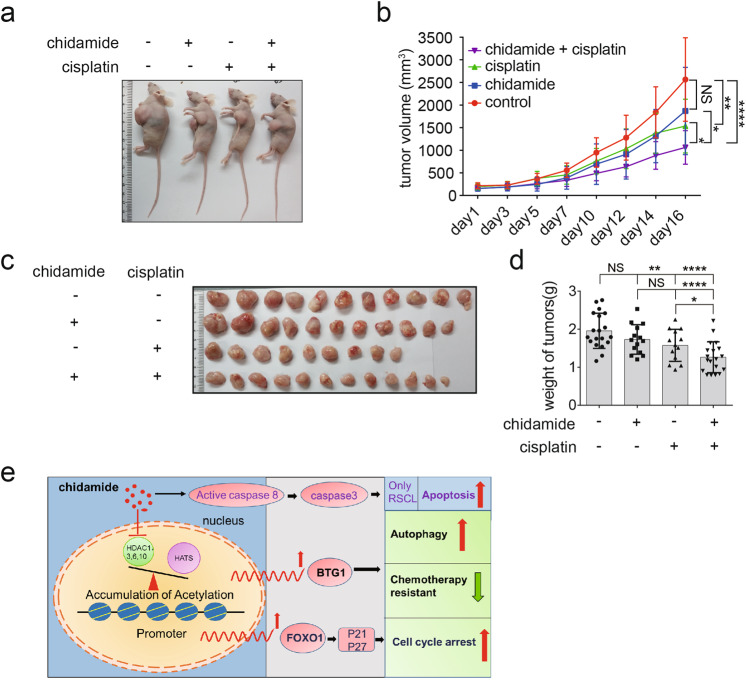
Chidamide and chemotherapeutics are synergetic in vivo. **a**–**d** Nude mice were subcutaneously with Raji-4RH cells in both flanks, after tumor volume is about 5 × 5 × 5 mm. These mice were randomly divided into 4 groups and treated with the combination of chidamide (25 mg/kg) and cisplatin (1 mg/kg) for about 2 weeks. Volumes of each group were shown in body (**a**) and growth curve (**b**). The weights **c** and sizes **d** of tumors were analyzed after sacrifice of mice. **e** Chidamide treatment caused the accumulation of histone acetylation at the promoter regions of FoxO1 and BTG1. Upregulation of FOXO1 increased the expression of p21 and p27, and induced cell cycle arrest. Above all, increased expression of BTG1 caused cell autophagy and decreased chemotherapeutics resistant (*N* = 3). For all graphs, data are presented as mean ± SD, *p < 0.05, **p < 0.01, *p* < 0.005***, *p* < 0.001****. Statistical analysis was performed with a paired *t* test.

The mouse model of the xenograft tumor was established by subcutaneous inoculation of 3 × 10^6^ rituximab-resistant Raji-4RH cells in nude mice. The latency of tumor formation to a volume of 5 × 5 mm at the site of injection was approximately 6–7 days after injection. The mice were randomly divided into 4 groups (Day 0) and treated with chidamide (2.5 mg/kg) in combination with cisplatin in the same way from Day 10 and tumor volumes were measured daily until Day 16. The tumor weights were examined at the endpoint of the experiment. Compared with the control group or chidamide/cisplatin single treatment group, the combination treatment significantly reduced the tumors sizes (*p* < 0.05) (Fig. 8a–c) and tumor weights (Fig. 8d). Moreover, the combination of chidamide and cisplatin showed increased anti-tumor effects compared with chidamide or cisplatin single treatment group. These results suggested that chidamide in combination with cisplatin showed synergistic therapeutic effect in the mouse model of rituximab-resistant B-cell lymphoma.

## Discussion

Overexpression of HDACs in tumor cells can induce proliferation and de­differentiation; conversely, knockdown of HDACs can induce a range of anti-tumor effects, including cell cycle arrest and inhibition of proliferation, induction of apoptosis, differentiation and senescence, and disruption of angiogenesis. This provides indication that HDAC inhibitors can be effective therapeutic drugs against cancers [24]. The patients with rituximab/chemotherapy relapsed and refractory B-cell lymphoma have low response rates to current available second-line treatment. We aimed to define the activity and biological effects of chidamide on the relapsed and refractory B-cell lymphoma. In this study, we explored chidamide’s anti-tumor activity in the rituximab-resistant pre-clinical models, and found that chidamide was active in RRCL and the primary relapsed B-cell lymphoma cells. Mechanistically, chidamide treatment increased the level of acetylated histone H3K9, which accumulated on the promoter regions of FOXO1/BTG1 and promoted their transcription activation in RRCL (Fig. 8e).

Chidamide-induced upregulation of FOXO1 resulted in the cell cycle arrest at G0/G1 phase, which were associated with the increase of cell cycle negative regulators p21/p27 and the decrease of cell cycle positive regulators E2F1/2, CDK2, CyclinA2, and CyclinB1. FOXO1 inhibition diminished chidamide activity in these cells suggesting that chidamide had FOXO1-dependent action on cell growth and cell cycle arrest.

Since the pro-apoptotic proteins are defective in RRCL, these cells cannot pass through apoptosis, the pro-apoptotic proteins–mediated programmed cell death. Chidamide exposure resulted in apoptosis in RSCL but not in RRCL, which indicated the existence of alternative pathways of caspase-independent cell death in RRCL. We previously demonstrated that loss of Caspase-3 increased the autophagy activation and elicited cytotoxic effects through an apoptosis-independent manner [25]. Both apoptosis and autophagy can be regulated by Caspase-3. RRCL is lack of Caspase-3, which may cause increased autophagy and apoptosis-independent cytotoxic effect upon stress condition. Autophagy has been shown to play a vital role in cell death, and some drugs such as arsenic trioxide, have been found to induce cell death via activation of autophagy [26, 27]. Upon the treatment of chidamide, RRCL chose the alternative manner of cell death, the BTG1-regulated autophagy. It has been shown that the levels of SQSMT1/p62 and LC3 are decreased in B-cell lymphoma compared to the reactive B cells, which indicates that the autophagy activity of B-cell lymphoma is increased [28–30]. Thus, autophagy may play a key role in the pathogenesis and treatment of B-cell lymphoma. BTG1 is a family member of anti-proliferative genes which regulate cell growth and differentiation [31]. As a cofactor and an adaptor molecule, BTG1 inhibits cell growth through transcriptional or post-transcriptional regulation [32]. In this study, we have found that chidamide-induced autophagy could be rescued by inhibition of BTG1.

Chidamide presented functional complementation with the chemotherapy drugs, through decreasing p62, thus promoting cell autophagy. The combination of chidamide with cisplatin sensitized the resistant cells to growth inhibition in a synergistic manner. More importantly, chidamide-cisplatin combination significantly blocked the growth of the tumor in a mouse lymphoma model established with engraftment of the rituximab/chemotherapy relapsed and refractory B-cell lymphoma cells. Therefore, chidamide in conjunction with cisplatin may represent a novel strategy in treating patients with rituximab/chemotherapy relapsed and refractory B-cell lymphoma. Taken together, chidamide is active in the rituximab-chemotherapy-resistant cell/mouse models with the induction of autophagy, and potentiates the antitumor activity of chemotherapy drugs, suggesting it has the potential of becoming an effective therapeutic agent in the treatment of rituximab/chemotherapy relapsed and refractory B-cell lymphoma. Our pre-clinical data supports further evaluation of chidamide in treating rituximab/chemotherapy relapsed and refractory B-cell lymphoma in a clinical trial.

## Supplementary information

supplementary figure legends

supplementary methods

S1

S2

S3

S4

S5

S6

## Data Availability

The data that support the findings of this study are available from the corresponding author upon reasonable request. The raw data used for the RNA-Seq are available in the Gene Expression Omnibus database under accession number GSE137359.
