# Supplementary material for: Chidamide triggers BTG1-mediated autophagy and reverses the chemotherapy resistance in the relapsed/refractory B-cell lymphoma
Source: Cell Death Dis. 2021 Oct 1;12(10):900. doi: 10.1038/s41419-021-04187-5 (PMC8486747; doi:10.1038/s41419-021-04187-5)
Supplement: Supplementary file 1 — supplementary figure legends [file 41419_2021_4187_MOESM1_ESM.docx]

**Supplementary Information**

**Figure S1. Chidamide treatment induced upregulation of acetylation of H3K9 in a dose dependent manner.**

Acetylation of H3K9 were examined by using western blot analysis in RRCL treated with chidamide (1 μM, 3 μM and 6 μM) for 48 hours (N=3).

**Figure S2. Chidamide treatment induced cell cycle arrest in both RSCL and RRCL, but triggered cell apoptosis only in RSCL.**

**a and b** The cell cycle of RSCL and RRCL treated with chidamide (1 μM, 3 μM and 6 μM) for 48 hours were examined by flow cytometry analyses **a**. The ratio of G1, S and G2/M phase were shown in histograph **b** (N=3). **c** Apoptosis of RSCL and RRCL induced by chidamide(1μM,3μM,6μM) treated for 48h were measured by flow cytometry. **d** Statistics of early apoptosis, late apoptosis and death were shown in corresponding histograph. (N=3). For all graphs, data are presented as mean ± SD.

**Figure S3. Cell cycle arrest molecular signature induced by chidamide treatment.**

**a** GSEA analysis of all genes shows a downregulation in HALLMARK E2F TARGETS genset after chidamide treatment compared with control, and the expression of p21, p27 were examined by using Q-PCR analysis in Raji or RL cells treated with chidamide for 24, 48 and 72 hours (N=3). **b** Downregulation in HALLMARK G2/M CHECKPOINT after chidamide treatment compared with the control, and the expression of E2F1, E2F2 and cyclinD3 were examined by using Q-PCR analysis in Raji or RL cells treated with chidmaide for 24, 48 and 72 hours (N=3). For all graphs, data are presented as mean ± SD, *p < 0.05, ** p < 0.01, p < 0.005***, p < 0.001****. Statistical analysis was performed with an unpaired *t* test.

**Figure S4. Upregulation of FOXO1 and changes of cell cycle related proteins induced by chidamide treatment.**

**a and b** Changes of p21, p27, CyclinA2, CDK2 and CyclinB1 were examined by Q-PCR and western blot analysis in RSCL and RRCL treated with chidamide (1 μM, 3 μM and 6 μM) for 48 hours (N=3). **c and d** Changes of FOXO1 were examined by using Q-PCR and western blot analysis in RSCL and RRCL treated with chidamide (1 μM, 3 μM and 6 μM) for 48 hours (N=3). For all graphs, data are presented as mean ± SD, *p < 0.05, ** p < 0.01, p < 0.005***, p < 0.001****. Statistical analysis was performed with an unpaired *t* test.

**Figure S5. Chidamide treatment induced robust autophagy in a concentration dependent manner.**

**a** RSCL and RRCL were treated with chidamide (1 μM, 3 μM and 6 μM) or DMSO control for 48 hours and autophagy related proteins were examined by using western blot analysis (N=3). **b** RSCL and RRCL were treated with Bafilomycin A1 (1 μM), chidamide (3 μM), chidamide (3 μM) and Bafilomycin A1 (1 μM) or DMSO control for 24,48 hours and autophagy related proteins were examined by using western blot analysis (N=3). **c and d** RRCL -EGFP-LC3 cells were treated with DMSO control, Bafilomycin A1 (1 μM), chidamide (3 μM) or their combination for 48 hours. EGFP fluorescence was measured by fluorescence microscope (Scale bar: 5 μm) and FACS (N=3) **c**. EGFP was examined by western blotting analysis (N=3) **d** . **e** Apoptosis of RRCL induced by Knock down BTG1 were examined by using flow cytometry analysis.The statistics of early apoptosis, late apoptosis were shown in corresponding histograph (N=3). For all graphs, data are presented as mean ± SD, *p < 0.05, ** p < 0.01, p < 0.005***, p < 0.001****. Statistical analysis was performed with an unpaired t test.

**Figure S6. Chidamide and chemotherapeutics were synergetic in RSCL.**

**a and b** Raji cells **a** and RL cells **b** were treated with the different combinations of chidamide with etoposide, cisplatin or hcl-gemcitabine for 72 hours and cell viability were examined by using MTT assay. Cooperativity of chidamide and chemotherapeutics were calculated by Compusyn software (N=3). **c** Raji-4RH and RL-4RH cells were treated with the different combinations of chidamide and Dox for 72 hours and cell viability were examined by using MTT assay. Cooperativity of chidamide and Dox were calculated by Compusyn software. The normalized isobologram were shown. DRI: Dose-Reduction Index (N=3). **d** The DNA damage and DNA repair related proteins were examined by Western blot analysi in Raji-4RH and RL-4RH cells treated with DMSO, chidamide (1 μM), cisplatin (15 μM) or their combination for 48 hours (N=3). For all graphs, data are presented as mean ± SD, *p < 0.05, ** p < 0.01, p < 0.005***, p < 0.001****. Statistical analysis was performed with an unpaired *t* test.
