# Supplementary material for: Chidamide triggers BTG1-mediated autophagy and reverses the chemotherapy resistance in the relapsed/refractory B-cell lymphoma
Source: Cell Death Dis. 2021 Oct 1;12(10):900. doi: 10.1038/s41419-021-04187-5 (PMC8486747; doi:10.1038/s41419-021-04187-5)
Supplement: Supplementary file 2 — supplementary methods [file 41419_2021_4187_MOESM2_ESM.docx]

**Reagents and antibodies**

The chemicals used in this experiments were: chidamide (Chipscreen, 450 Caoyang Road, Shanghai), cisplatin (qilu-pharma, 8888 Tourism Road, Gaoxin District, Jinan City), hcl-gemcitabine (hansoh, 9 Dongjin Road, Huaguoshan Dadao, Lianyungang City), etoposide (HENGRUI-pharma, 7 Kunlun Shan Road, Lianyungang Economic and Technological Development Zone), MTT (Amresco, 0793, 30175 Solon Industrial Parkway) and AS1842856 (AS184) (MCE, 836620-48-5, Lane 1999, Zhangheng Road, Pudong New Area, Shanghai), Propidium iodide (PI) (Sigma, P4170, Lane 227, Dongyu Road Pudong New District Shanghai), matrigel (bd biocoat, 354234, Lane 166, Tianxiong Road, Pudong New Area, Shanghai) and APC-Annexin V (BD Pharmingen, 550474, 75 Xiilie Middle Road, Yuexiu District, Guangzhou). The antibodies used in our experiments: GAPDH (proteintech, 354358, 666 Gaoxin Avenue, East Lake New Technology Development Zone, Wuhan), LC3B (Novus Biologicals, NB100-2220, Briarwood Avenue, CO 80112, USA), histone H3 (Cell Signaling Technology, 4499, 399 Shengxia Road, Pudong New Area, Shanghai), Acetyl-Histone H3 (Lys9) (Cell Signaling Technology, 9649), p62 (Cell Signaling Technology, 39749S), CyclinA2 (Cell Signaling Technology, 4656), CyclinB1 (Cell Signaling Technology, 12231T) and Cell Cycle Regulation Antibody Sampler Kit (Cell Signaling Technology, 9932T).

**Cell Viability assay (MTT assay)**

RSCL, RRCL and patient samples were plated at a cell density of 1 × 10^5^ cells/mL in 96 well plates (50 µL/well) and were exposed to different concentrations of chidamide, etoposide, cisplatin and hcl-gemcitabine, either alone or in combinations. 0.1 mg MTT was added to each well. After the incubation at 37°C for 4 hours, formazan was decomposed by 50 µL triple lysis buffer (10 % SDS, 5 % isopropanol and 0.012M HCL) overnight and then the absorbance was measured at 562 nm by spectrophotometry.

**Isobolographic analysis**

Determination of the synergistic of etoposide, cisplatin, hcl-gemcitabine, Dox and chidamide was performed by the combination index (CI) method described by Chou and Talalay (CalcuSyn software, Biosoft). When at least 80 % of CI values for a combination were less than one, the drug combination was considered to be synergistic.

**Lentivirus production and infection**

For the production of lentivirus, 293T cells were transfected with gene plasmids (PIG, shBTG1-1 or shBTG1-2) and packaging plasmids (pspax2 and pMD2G) by calcium phosphate-DNA coprecipitation. Virus supernatants were collected at 48, 72 and 96 hours after transfection and filtered with 0.45 μM sterile millex filters. 5 x 10^5^ Raji-4RH cells were infected with 1 mL fresh filtered virus supernatant and 8 μg/mL of Polybrene (Sigma), then centrifuged at 1500 rpm for 60 minutes at room temperature. The medium was changed 12 to 20 hours after virus infection.

**ShRNA mediated gene knockdown**

The BTG1 shRNAs were constructed with shRNA-sequences from GPP Web Portal. Virus preparation and cell infection were performed as described above. The efficiency of knockdown were measured by Q-PCR 4 days after infection. The sequences of shRNA used were given in supplementary Table 1.

**RT-PCR and RT-qPCR**

The total RNA was extracted with TRIzol Reagent (Invitrogen) and cDNA was synthesized by PrimeScript™ RT reagent Kit with gDNA Eraser (TaKaRa #RR047A). SYBR® Premix Ex Taq™ GC (ROCHE) was used for quantitative Real-time-PCR (RT-qPCR) analysis, Glyceraldehyde-3-phosphate dehydrogenase (GAPDH) was used as an internal control of RNA integrity. RT-qPCR was performed in triplicate. The sequences of primers used were given in supplementary Table 1.

**ChIP-qPCR**

ChIP assay was carried out as the method of Lan et al ^1^. Raji-4RH was treated with chidamide (3 µM) for 24 hours at a cell density of 5 x 10^5^/ml. Then 2 x 10^7^ DMSO and chidamide treated Raji-4RH were crosslinked with 1 % formaldehyde for 10 minutes and then stopped by 125 mM Glycine for 5 minutes. After lysis and sonication, each sample was incubated with 30 µL protein A/G beads (Smart life sciences SA032005) at 4 ℃ for 1 hour and then divided into 2 parts. 5 μg of Acetyl-Histone H3K9 (Cell Signaling Technology, 9649) Rabbit antibody and 5μg IgG antibody were incubated with chromatin sample overnight at 4℃. Next, the samples were incubated with 50 μL protein A/G beads for 2 hours. Finally, DNA were purified with PCR recovery kit (QIAGEN #28006), and then the amount of desired DNA fragments were measured with Q-PCR. The sequences of primers used were seen in supplementary Table 1.

**RNA-seq**

Raji and Raji-4RH were treated with chidamide (3 µM) for 24 hours at a cell density of 5 x 10^5^/mL and RNA was extracted with TRIzol Reagent (Invitrogen). A total amount of 3 µg RNA per sample was used as input material for the RNA sample preparation. Sequencing libraries were generated using NEBNext® UltraTM RNA Library Prep Kit for Illumina® (NEB, USA) following manufacturer’s recommendations and sequenced with Illumina HiSeq X ten.

**Flow cytometry**

All the flow cytometry experiments were performed on the Beckman Coulter cell analyzer and analyzed with FlowJo V10 or MFLT32 for cell cycle analysis. For apoptosis analysis, 1 × 10^5^ cells were incubated at room temperature for 15 minutes in 1 × binding buffer with APC Annexin V and propidium iodide (PI, 10 µg/mL) , and then analyzed on a Beckmann Gallios cell analyzer machine. For cell cycle analysis. For cell cycle analysis, 1 × 10^6^ cells were fixed with 70 % Alcohol PBS over 24 hours at -20 ℃, and then disposed with RNAase (100 µg/mL) for 3 0min at 37℃. The cells were washed once with 1 × PBS and incubated with PI (10 µg/ml) for 15 min.

**Statistical Analysis and Bioinformatics**

Isobolographic analysis: determination of the synergistic of etoposide, cisplatin, hcl-gemcitabine and chidamide was performed by the combination index (CI) method described by Ting-Chao Chou and Nick Martin (ComboSyn software). When at least 80% of CI values for a combination were less than one, the drug combination was considered to be synergistic. Bioinformatics: the GSEA v2.0 software (http://www.broad.mit.edu/gsea) was used to perform gene set enrichment analysis (GSEA) ^2^. All other statistical analyses were performed using GraphPad Prism software. Unpaired student’s t test was used for comparison of groups of samples of equal variance. The IC50 was calculated using GraphPad Prism software as following Open GraphPad software, select "XY", then click "Create".Click “Analysis” and choose “Transform concentrations (X)” select “Transfom to logarithms”.Click “Analysis” again, then click the "Nonlinear Regression (Curve Fit)" and select "Log (Inhibition) vs. Normalized Response -- Variable Slope".Click “OK” to get the calculated IC50.

1. Lan F, Collins RE, De Cegli R, Alpatov R, Horton JR, Shi X*, et al.* Recognition of unmethylated histone H3 lysine 4 links BHC80 to LSD1-mediated gene repression. *Nature* 2007, **448**(7154)**:** 718-722.

2. Subramanian A, Tamayo P, Mootha VK, Mukherjee S, Ebert BL, Gillette MA*, et al.* Gene set enrichment analysis: A knowledge-based approach for interpreting genome-wide expression profiles. *Proceedings of the National Academy of Sciences of the United States of America* 2005, **102**(43)**:** 15545-15550.
