# Supplementary figures and images for: Chidamide triggers BTG1-mediated autophagy and reverses the chemotherapy resistance in the relapsed/refractory B-cell lymphoma
Source: Cell Death Dis. 2021 Oct 1;12(10):900. doi: 10.1038/s41419-021-04187-5 (PMC8486747; doi:10.1038/s41419-021-04187-5)

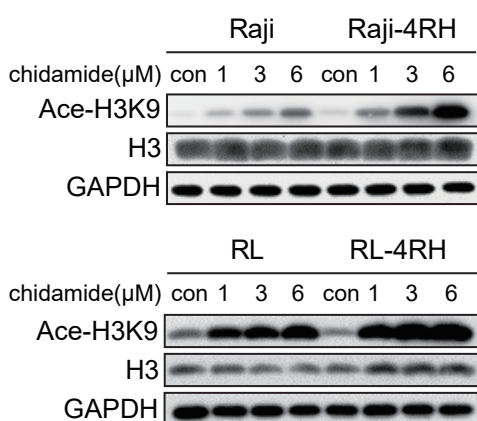

Figure S1

Supplement: Supplementary file 3 — S1 [file 41419_2021_4187_MOESM3_ESM.pdf]

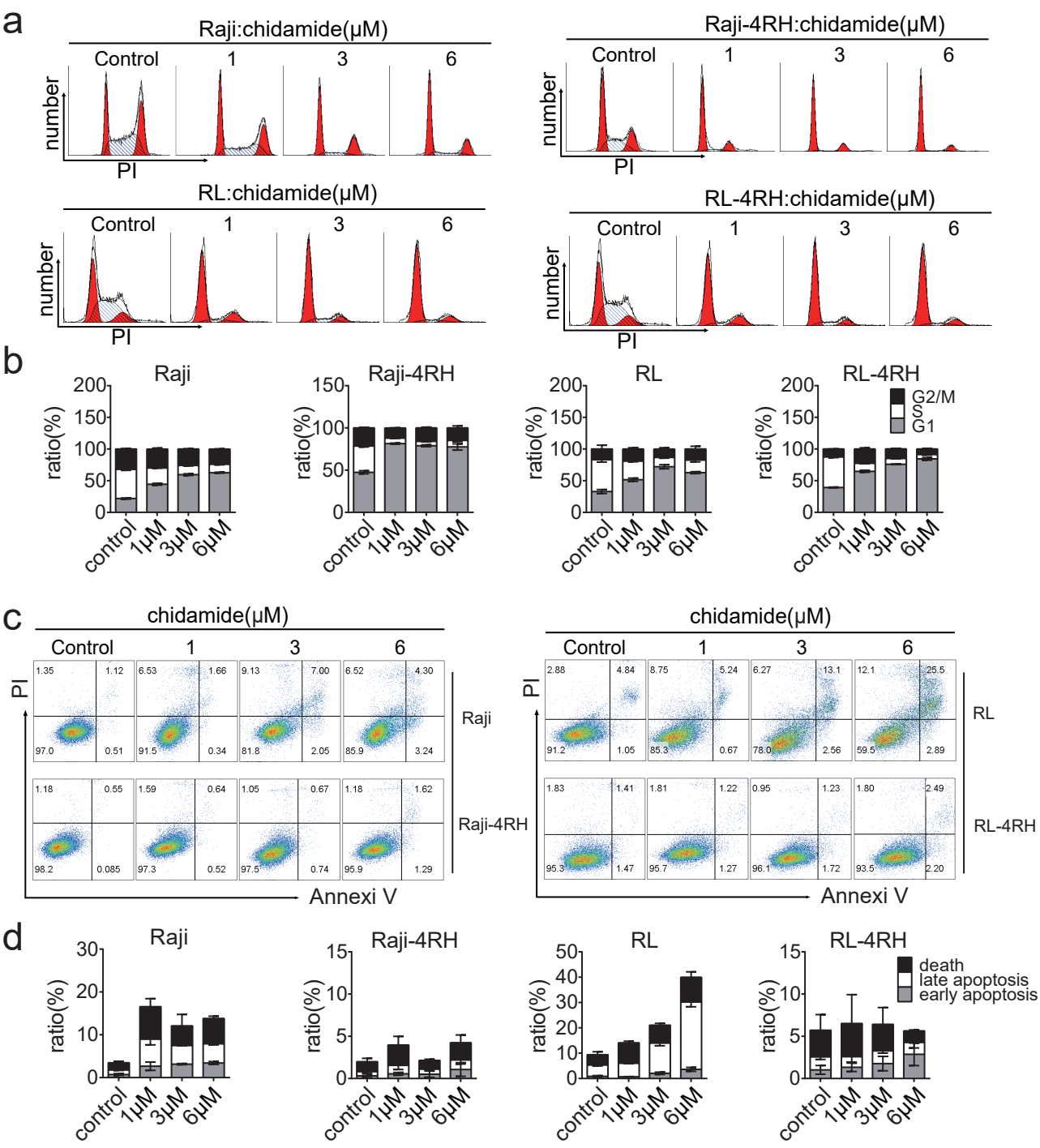

Figure S2

Supplement: Supplementary file 4 — S2 [file 41419_2021_4187_MOESM4_ESM.pdf]

a

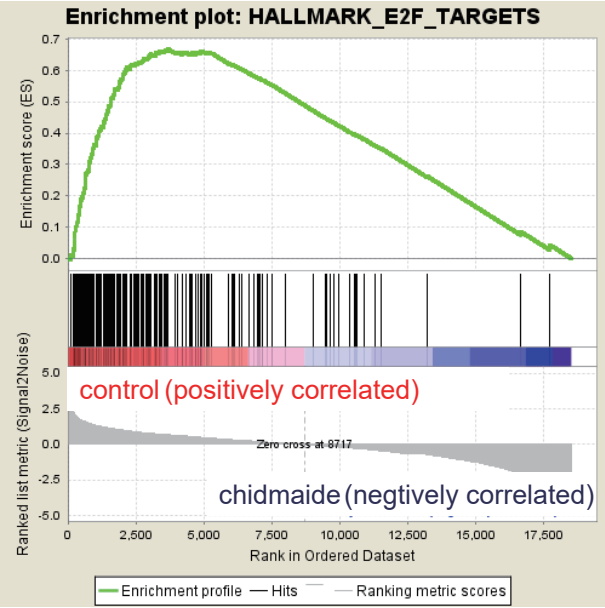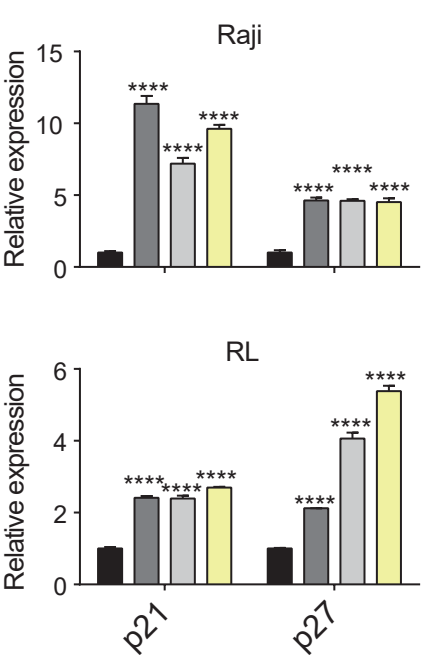

b

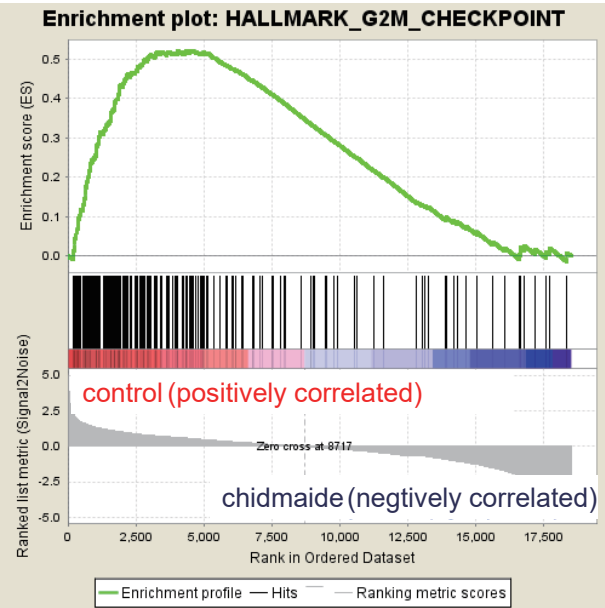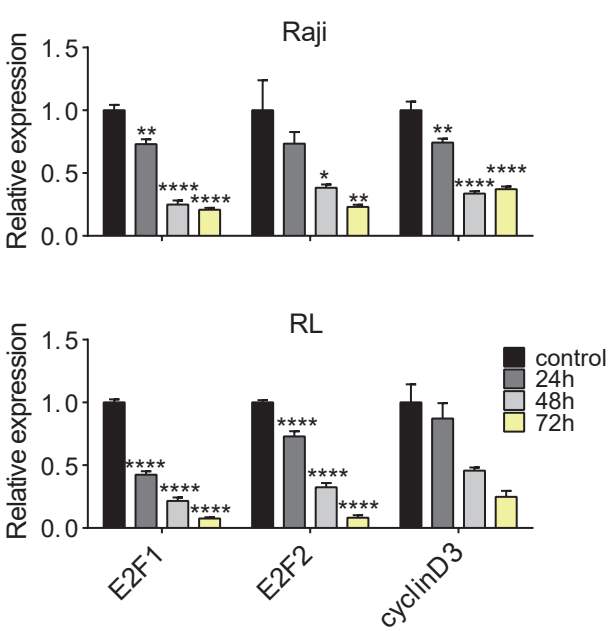

Figure S3

Supplement: Supplementary file 5 — S3 [file 41419_2021_4187_MOESM5_ESM.pdf]

a

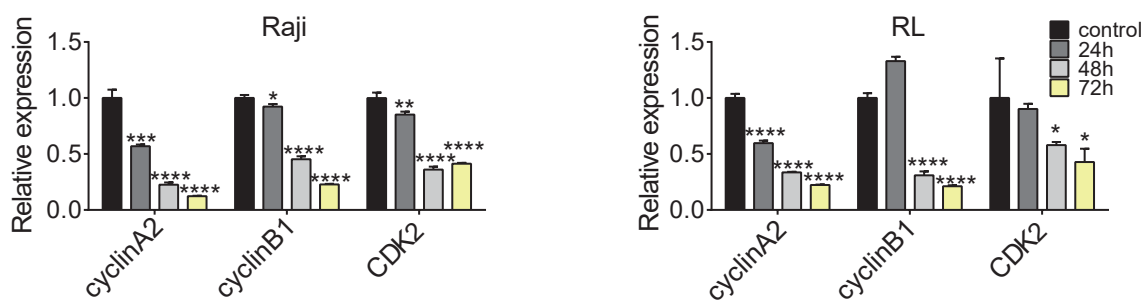

b

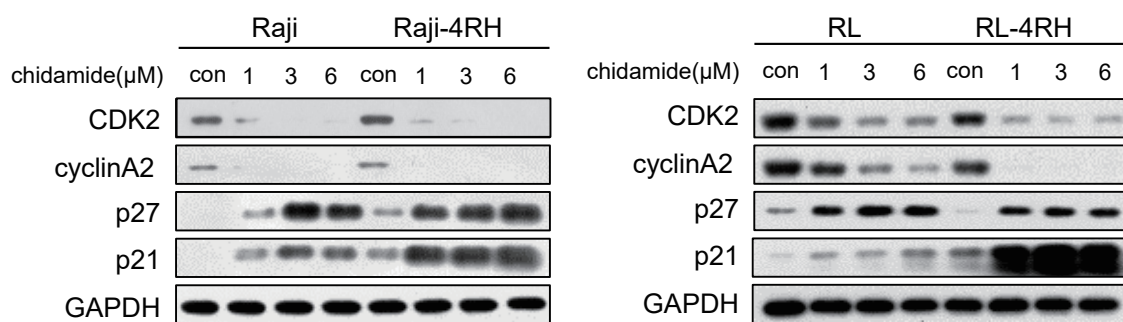

c

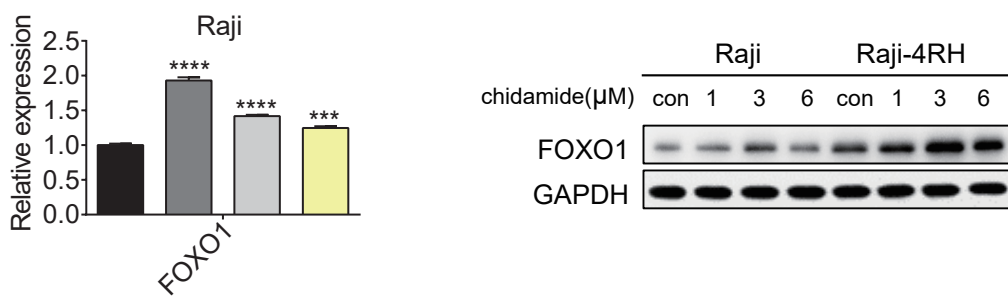

d

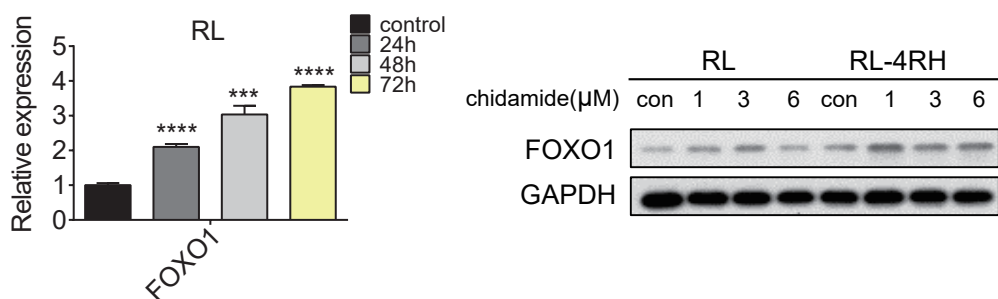

Figure S4

Supplement: Supplementary file 6 — S4 [file 41419_2021_4187_MOESM6_ESM.pdf]

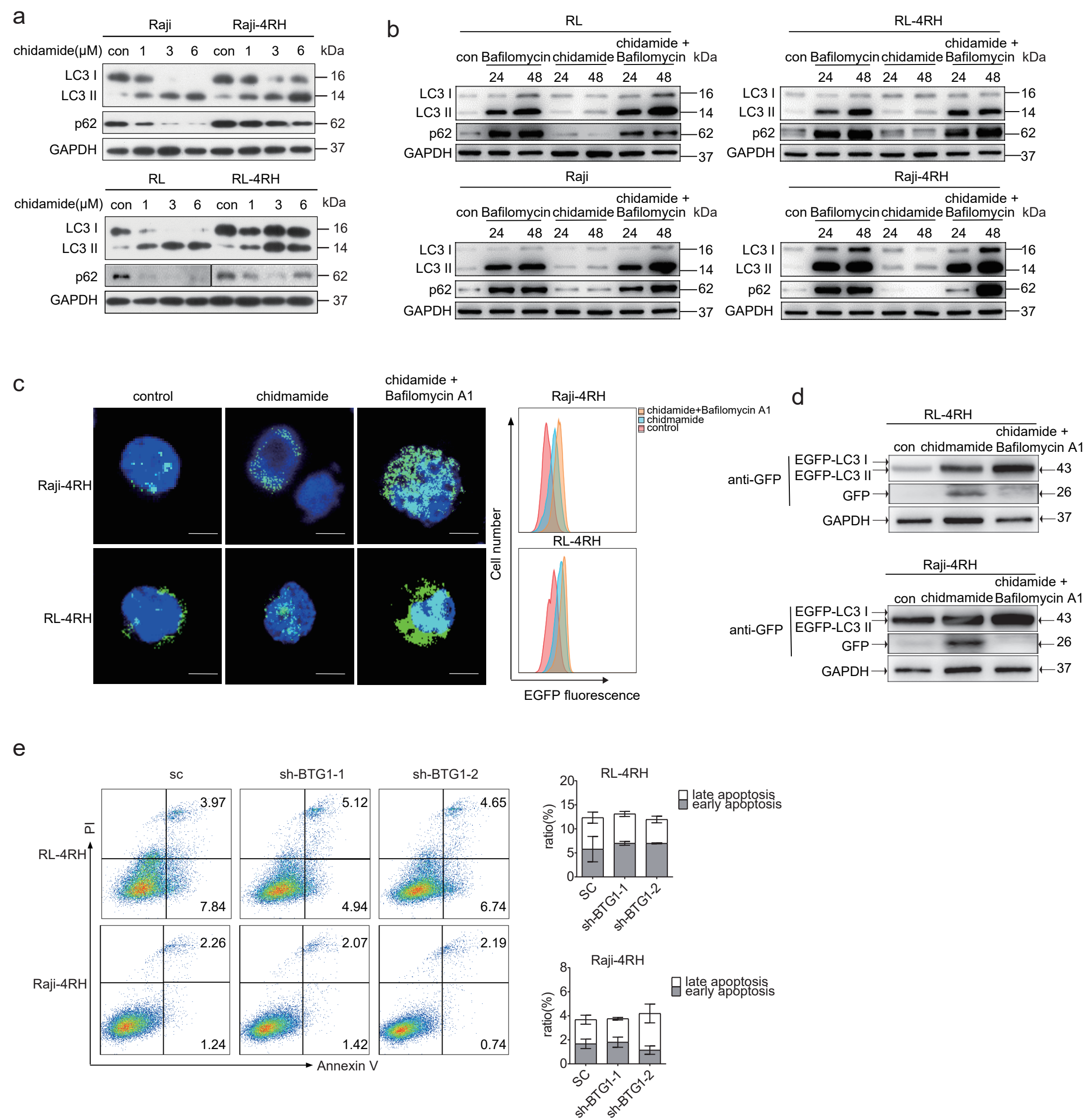

Figure S5

Supplement: Supplementary file 7 — S5 [file 41419_2021_4187_MOESM7_ESM.pdf]

a

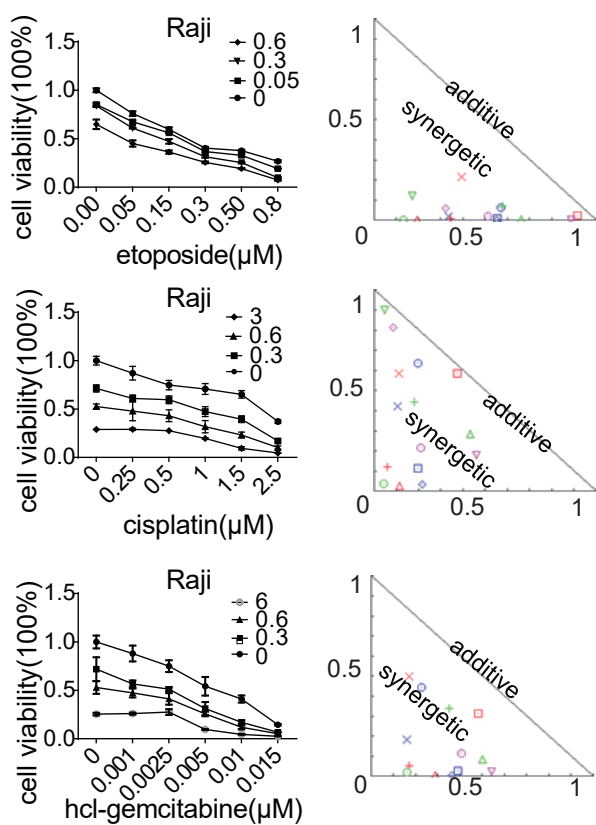

b

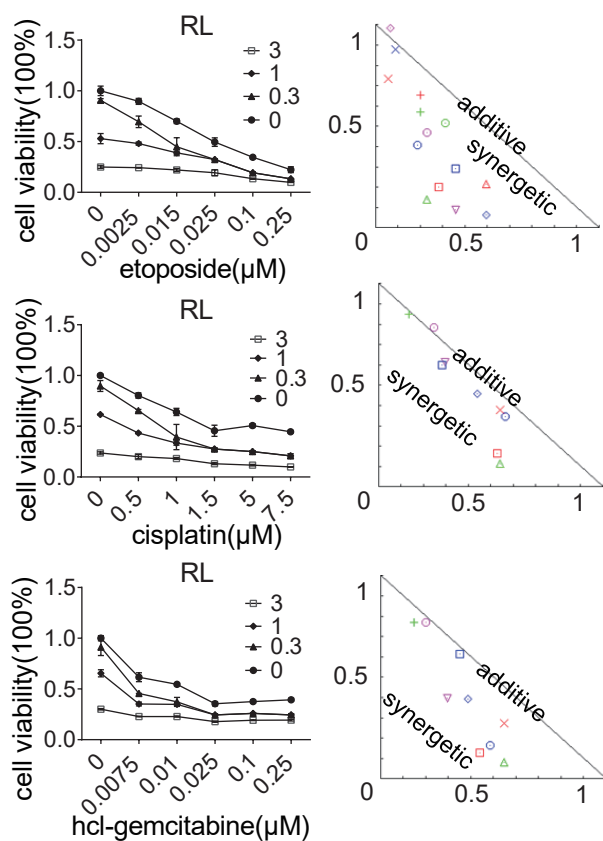

c

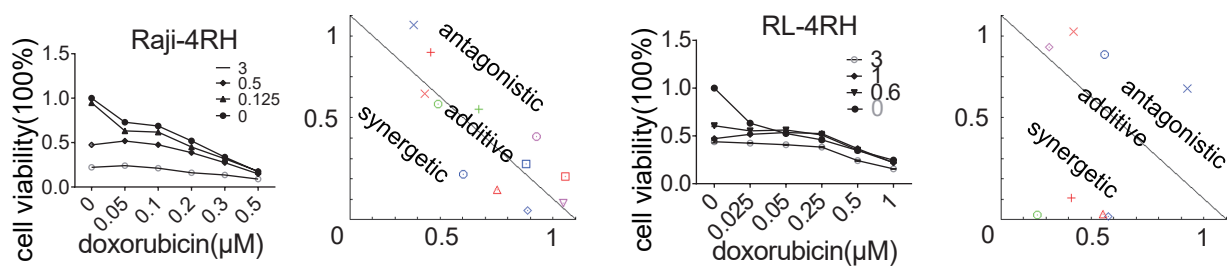

d

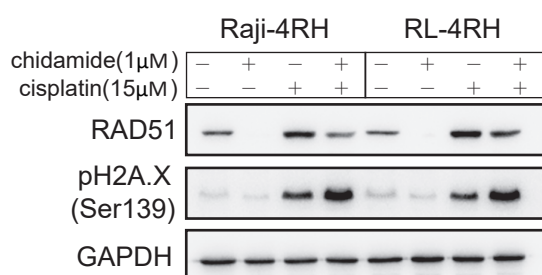

Figure S6

Supplement: Supplementary file 8 — S6 [file 41419_2021_4187_MOESM8_ESM.pdf]
